# Impact of Urban Residents’ Environmental Cognition on Voluntary Carbon-Reduction Behavior: The Mediating Role of Environmental Emotion

**DOI:** 10.3390/ijerph192315710

**Published:** 2022-11-25

**Authors:** Ting Yue, Qianru Wang, Ruyin Long, Hong Chen, Mengting Li, Haiwen Liu

**Affiliations:** 1 School of Economics and Management, China University of Mining and Technology, Xuzhou 221116, China; 2 School of Business, Jiangnan University, Wuxi 214122, China; 3 The Institute for Jiangnan Culture, Jiangnan University, Wuxi 214122, China; 4 Research Institute of National Security and Green Development, Jiangnan University, Wuxi 214122, China

**Keywords:** voluntary carbon-reduction behavior, environmental emotion, environmental cognition

## Abstract

Urban residents play an essential role in the carbon-reduction process, and it is significant to effectively guide them to reduce carbon voluntarily to achieve the “double carbon” target. In this study, a model was developed to investigate the influence of the environmental cognition (EC), environmental emotion (EE), and voluntary carbon-reduction behavior (VCB) of urban residents. Based on a sample of 978 urban residents in Jiangsu province, we used a multiple regression analysis to investigate the mechanisms of EC and EE on VCB. The results showed that: (1) both EC and EE positively affected residents’ VCB, and EC had a higher impact than EE; (2) the three dimensions of EC (cognition for carbon-reduction knowledge, cognition for environmental issues, and cognition for individual responsibility) and the two dimensions of EE (positive environmental emotion and negative environmental emotion) all had a significant positive effect on voluntary carbon-reduction behavior; and (3) EE played a partial mediating role in the relationship between EC and VCB, and there was no significant difference in the strength of the mediating effect between positive and negative environmental emotion. In conclusion, raising environmental awareness and stimulating environmental emotion have the potential to promote voluntary carbon-reduction behavior among residents.

## 1. Introduction

China is the world’s largest energy consumer and carbon emitter, which accounts for one-third of global CO_2_ emissions; the pace of China’s emissions reduction has become an essential factor in controlling global warming within 1.5 °C [1]. Currently, China’s carbon-reduction efforts mainly focus on the corporate sector, such as electricity and coal, with less attention paid to the household sector as an essential carbon-reduction sector [2]. As prominent participants in household carbon emissions, urban residents play a vital role in the carbon-reduction process. Stimulating and improving residents’ enthusiasm and willingness to reduce carbon is conducive to alleviating the pressure of carbon reduction in life. However, Chinese urban residents still have the phenomenon of “high awareness and low degree of practice” when it comes to ecological environment behavior and cannot achieve “the unity of knowledge and action” [3]. Therefore, in the context of double carbon, it is imperative to explore the relationship between residents’ environmental perception and voluntary carbon-reduction behavior and to effectively guide their voluntary choice of carbon-reduction behavior.

At present, many scholars have explored the influence of environmental cognition on pro-environmental behavior from different perspectives. Some scholars have directly explored the influence of environmental cognition on pro-environmental behavior [4,5]. Other scholars have classified environmental cognition into dimensions such as perception of climate change [6], personal environmental awareness, and environmental knowledge [7,8] to explore the effects of different types of environmental cognition on pro-environmental behavior. With the development of environmental behavior research, it has been found that the association between the level of individual environmental cognition and actual pro-environmental behavior is not significant [9], and the limitations of the influence of cognitive factors on environmental behavior are increasingly evident. Scholars found an intrinsic link between cognition, emotion, and physical behavior [10]. They began to explore the relative ability of environmental cognition and emotion under different environmental behaviors, such as green purchase behavior [11], green consumption behavior [12], public pro-environmental behavior [13], and travelers’ pro-environmental behavior [14]. However, we found that not much of the literature has been devoted to systematically exploring residents’ carbon-reduction behaviors, and the voluntary character of residents’ carbon-reduction was rarely mentioned.

Therefore, this paper aimed to investigate the mechanism of the environmental cognition and environmental emotion of urban residents on voluntary carbon-reduction behavior and to examine the mediating role of environmental emotion between environmental cognition and voluntary carbon-reduction behavior. It provides a reference for the government and relevant departments to guide and promote urban residents to implement voluntary carbon-reduction behavior.

## 2. Theoretical Background and Hypotheses

### 2.1. Theoretical Model

The knowledge–attitude–practice (KAP) model believes that individual behavior change is a process, and there are three processes: knowledge (cognition), attitude (belief), and practice (behavior) [15]. This model was widely used in individual behavior research [16,17,18]. Wang et al. [19] proposed an extended knowledge–attitude–practice (EKAP) model, which expanded “knowledge” to “cognition and perception” and “attitude” to “emotion and consciousness”, effectively explaining the mechanism of psychological consciousness factors influencing individual ecological civilization behavior. Ye [12] further expanded on the model by examining the mechanism of green cognition and emotion on environmentally friendly green consumption behavior. Based on this, we developed a framework model of urban residents’ environmental perception, environmental emotion, and voluntary carbon-reduction behavior. Since the voluntary carbon-reduction behavior of urban residents is different from general pro-environmental behavior, it was necessary to expand the scope and redefine the concept of cognition, emotion, and behavior in the context of urban residents and then comb through the relevant literature and formulate specific research hypotheses.

#### 2.1.1. Environmental Cognition (EC)

Cognition is the process of acquiring knowledge and making sense of events [20]. In this paper, environmental cognition (EC) was defined as the degree of urban residents’ perception of their environment and carbon-reduction knowledge and their perception of the responsibility for carbon-reduction. Some scholars have classified EC to study its influence on pro-environmental behavior. Most of the literature focused on two areas: one was knowledge of systemic aspects such as natural laws and ecological balance [21]; the other was individuals’ perception of the severity, urgency, and personal relevance of environmental degradation problems [22]. In addition, this study considered the human social characteristics of urban residents, whose sense of moral responsibility as social citizens cannot be ignored. Therefore, on the basis of previous research, this paper divided EC into three dimensions: cognition for environmental issues (CEI), cognition for carbon-reduction knowledge (CCK), and cognition for individual responsibility (CIR). CEI refers to the individual’s perception of the seriousness and urgency of the environmental degradation problem; CCK refers to the individual’s knowledge of the meaning of carbon reduction and carbon-reduction behavior guidelines; and CIR refers to the individual’s sense of responsibility and mission to adjust their high-carbon behavior in response to the environmental degradation problem.

#### 2.1.2. Environmental Emotion (EE)

Environmental emotion is the individual’s attitudinal experience of whether a carbon-reduction-related issue or behavior meets their needs. It can be either a reflection of positive attitudes (e.g., love, approval, pride) or negative attitudes (e.g., worry, shame, disgust) [23,24]. Personal feelings about the environment should include emotion for different subjects, including feelings about the environmental situation and one’s or others’ environmental behavior. Thus, this paper defined environmental emotion (EE) as an attitudinal experience that residents have about whether or not they or others are performing carbon-reduction behaviors. We divided EE into two dimensions: positive environmental emotion (PEE) and negative environmental emotion (NEE). PEE refers to the positive psychological states of individual residents, such as love, praise, and pride for low-carbon behaviors in life; NEE refers to the negative psychological state of individual residents, such as worry, guilt, and disgust.

#### 2.1.3. Voluntary Carbon-Reduction Behavior (VCB)

As a subset of environmental behavior, carbon-reduction behavior is the way in which residents act to protect the living environment and use resources wisely to balance environmental protection with their daily lives. As a kind of pro-environmental behavior, voluntary carbon-reduction behavior adds the initiative of the implementer to the carbon-reduction behavior, in which individuals take the initiative to solve environmental problems through conscious behavior. Therefore, this paper defined voluntary carbon-reduction behavior (VCB) as a conscious effort by urban residents to conserve resources, protect the environment, and choose a long-term lifestyle low in energy consumption, low in pollution, and conducive to healthy urban development.

### 2.2. Research Hypothesis

#### 2.2.1. The Impact of EC on VCB

Individual perception and behaviors are inseparable, and people can change their perception to correct irrational behaviors by changing their perception. Psychological factors such as cognition play an important role in environmental protection, and they change people’s behavior in a subtle and long process, thereby prompting people to protect their living environment [25]. If residents voluntarily practice carbon reduction in their daily lives, they need to develop the appropriate perception in their knowledge structures. As a typical pro-environment behavior, we have reason to believe that EC plays an important role in the VCB. In addition, different from general pro-environmental behaviors, VCB reflects the initiative of the actors, so EC may have a more critical impact on it.

First of all, CEI is a prerequisite for generating environmental behavior motivation and implementing environmental behavior. Regarding the relationship between CEI and individual carbon-reduction behavior, scholars’ research results were relatively consistent, and they believed there was a positive relationship between CEI and individual carbon-reduction behavior [26]. When the public realizes that the environmental status quo may bring risks, they are more likely to pay attention to improving environmental quality and adopt more pro-environmental behaviors. Therefore, it is reasonable to assume that individuals with a higher awareness of current environmental issues will voluntarily implement more carbon-reduction behaviors.

Secondly, scholars generally believed there was a positive correlation between CCK and environmental behavior [27]. Individuals with rich environmental knowledge will be more likely to adopt more pro-environmental behaviors [28]. CCK was generally considered to be one of the critical factors affecting individual carbon-reduction behavior [29]. Therefore, this study identified CCK as a driving factor for urban residents’ VCB.

Finally, the normative behavior theory model believes that when actors realize that their behavior may have positive consequences for themselves, they will have a sense of behavioral responsibility and implement the behavior [30]. Individuals with a sense of responsibility are more aware of their responsibilities and, therefore, more rational in making behavioral choices. Therefore, we believed that the degree of CIR would affect the VCB of its residents. Hence, we proposed the following assumptions:

**H1.**  *EC positively affects residents’ VCB.*

**H1-1.**  *CEI positively affects residents’ VCB.*

**H1-2.**  *CCK positively affects residents’ VCB.*

**H1-3.**  *CIR positively affects residents’ VCB.*

#### 2.2.2. The Impact of EE on VCB

The research conclusions on the impact of environmental emotion on low-carbon behavior are basically consistent, and there is a positive correlation between environmental emotion and low-carbon behavior [31]. According to Affective Event Theory (AET), when individuals experience positive or negative events in their lives, these events will trigger their emotional response, which further affects their behavioral willingness after individual self-representation, self-awareness, and self-evaluation [32]. In voluntary carbon reduction, when people perceive environmental degradation, they feel guilty and sad about their own or others’ non-environmental behavior, which motivates residents to actively participate in carbon reduction. At the same time, experiencing the comfort of the natural environment or receiving compliments for performing carbon reduction behaviors will also encourage residents to engage in carbon reduction behavior again. Therefore, we concluded that individuals’ emotional experiences of whether or not to perform carbon-reduction behaviors could significantly and positively influence their VCB. Therefore, we proposed the hypotheses as follows:

**H2.**  *EE positively affects residents’ VCB.*

**H2-1.**  *PEE positively affects residents’ VCB.*

**H2-2.**  *NEE positively affects residents’ VCB.*

#### 2.2.3. The Mediating Effect of EE

Emotion is the emotional reflection of the individual after a cognition, which can reflect the emotion and feelings contained in the individual’s attitude, while behavior shows the individual’s action intention [33]. Cognition is the antecedent variable of emotion, that is, the individual’s cognition of external things and stimuli will generate related emotion and then generate behaviors. When people confront environmental problems, their perception of the environment stimulates their environmental emotion, which in turn influences the occurrence of pro-environmental behavior. The influence of cognitive activities on pro-environmental behavior does not occur directly but is mediated by emotional factors [34]. For residents’ VCB, when the individual has the corresponding environmental cognition, facing severe environmental problems, it will induce residents’ feelings for environmental protection and then promote the occurrence of carbon-reduction behavior. Based on this, we argued that residents’ EE may not only directly affect VCB but may also serve as a mediating variable. Therefore, we proposed the following assumptions:

**H3.**  *EE mediates the effect of EC on residents’ VCB.*

**H3-1.**  *PEE mediates the effect of EC on residents’ VCB.*

**H3-2.**  *NEE mediates the effect of EC on residents’ VCB.*

**Figure 1:**
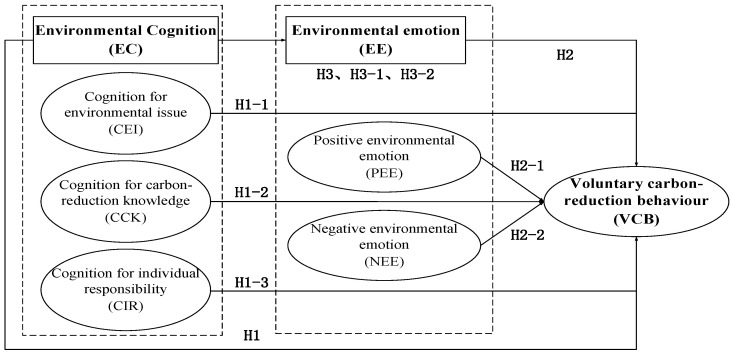
Conceptual model.

Based on the above theoretical foundation and research hypotheses, we constructed a theoretical model of the influence of urban residents’ EC and EE on VCB, as shown in Figure 1.

## 3. Materials and Methods

### 3.1. Participants and Procedure

In this study, a simple sample was used to select urban residents from 13 cities in Jiangsu Province as the primary research subjects, including Nanjing, Xuzhou, Suzhou, and other cities. By distributing electronic questionnaires, a total of 1056 questionnaires were collected, and excluding invalid questionnaires, 978 valid questionnaires were finally obtained, with a valid return rate of 92.6%.

The results of the sample characteristics of the questionnaire are shown in Table 1. Among them, 51.2% were male and 48.8% were female. Participants’ age levels were: 18–25 (n = 159,16.26%), 26–35 (n = 468, 47.85%), 36–45 (n = 267, 27.30%), 46–60 (n = 75, 7.67%), and older than 60 (n = 9, 0.92%). Participants’ education levels were: high school and below (n = 253, 25.87%), college (n = 298, 30.47%), bachelor’s degree (n = 336, 34.36%), and master’s degree and above (n = 91, 9.30%). Participants’ income levels were: less than RMB 2000 (n = 122, 12.48%), RMB 2000–6000 (n = 352, 35.99%), RMB 6000–10,000 (n = 405, 41.41%), and more than RMB 10,000 (n = 99, 10.12%).

### 3.2. Measures

This paper measured residents’ EC, EE, and VCB through questionnaires and collected the respondents’ demographic information such as gender, age, and education level. The independent variable EC included three dimensions: CEI, CCK, and CIR. The CEI was referenced to Knussen et al. [35] and contained four items; the CCK was referenced to Wu et al. [36] and contained four items; and the CIR was referenced to Whitmarsh et al. [7] and contained three items. For the dependent variable, VCB, this paper referred to the scale design of Stern [37] and Chen et al. [38] for general pro-environmental behaviors. Based on this, the scale was self-developed by combining the unique characteristics of voluntary carbon-reduction behaviors and ended up containing a total of seven question items. Emotions can be classified as positive and negative. In this paper, the mediating variable EE was measured in PEE and NEE. With reference to the studies of Palmer et al. [39] and Wang [40], three question items were set for each of the two dimensions, PEE and NEE, respectively. A Likert 5-point score was used for the measurement of the above variables (“completely disagree” = 1, “completely agree” = 5), and the specific scales are shown in Table 2.

### 3.3. Reliability and Validity Test of Scales

The reliability of the scale was tested by SPSS 26.0. Cronbach’s α coefficient of each variable was above 0.65 (Table 3), indicating that the reliability of the questionnaire was good [41]. Confirmatory factor analysis (CFA) was conducted by AMOS 24.0 to ensure the validity of the research scale. The standardized factor load of each item was greater than 0.5, the combined reliability (CR) was greater than or close to 0.7, and the average variance extraction (AVE) was greater than 0.4 (Table 2), indicating that the convergent validity of the variables in this study was within the acceptable range [42].

A validation factor test was conducted by AMOS 24.0 to compare the fit of the original, five-factor, four-factor, three-factor, and one-factor models to verify the discriminant validity between the factors. As seen in Table 4, the original model was the best. RMSEA < 0.08, GFI, and CFI were all above 0.9, and χ^2^/df < 5. The other models were worse for each fit indicator compared to the original model and passed the chi-square test at a significance level of 0.001, indicating good model discriminant validity [43].

### 3.4. Common Method Bias

This study used Harman’s one-way test to analyze the issue of common method bias for each variable to avoid common method bias. The results showed that the unrotated factor analysis yielded three factors with a characteristic root greater than one, and the first factor explained 38.815% of the variance, which was less than the 40% threshold, so there was no serious common method bias in this study [44].

## 4. Results

### 4.1. Correlation Analysis

Table 5 illustrates the investigated variables’ means (M), standard deviations (SD), and correlations. The results of the correlation analysis showed a two-by-two significant positive correlation between the dimensions at the 0.01 level of significance. A comparison of the mean values of the EC dimensions showed that the mean value of CIR was higher than that of CEI and CCK. It indicated that residents had a high level of individual responsibility, but their knowledge of environmental issues and carbon reduction needed to be improved. In addition, the mean values of all dimensions of EE were larger than those of EC, indicating that urban residents in Jiangsu Province had a higher environmental protection emotion compared to the environmental perception.

### 4.2. Difference Analysis

As shown in Table 6, gender significantly differed in individual responsibility cognition (*p* < 0.05), and the female individual responsibility cognition score was significantly higher than that of the male. Age showed a significant difference in VCB (*p* < 0.05) and CCK (*p* < 0.01), respectively. Both VCB and CCK showed an inverted ‘U’ shape with age. Income also had a significant difference in VCB, CEI, and CCK, with those earning over RMB 2000 having a higher VCB, CEI, and CCK than those earning less than RMB 2000.

### 4.3. Hypothesis Testing

#### 4.3.1. The Main Effect of Each Dimension on VCB

After relevant analysis, in order to further analyze the influencing factors of residents’ VCB, a multiple regression model was used to investigate the impact of environmental cognition and emotion on VCB. In the regression model, we first considered the effects of EC and EE on VCB (Model 1) and then considered the effects of each dimension of EC and EE on VCB (Model 2). We used SPSS 26.0 to test the model’s hypothesis, and the results are shown in Table 7.

For Model 1, it can be seen that both EC (β = 0.616, *p* < 0.001) and EE (β = 0.357, *p* < 0.001) had a significant positive effect on residents’ VCB, and the effect of EC was much higher than that of EE. Thus, H1 and H2 were verified. Model 2 showed that the regression coefficients between the dimensions of EC (CEI, CCK, and CIR) and residents’ VCB were 0.20, 0.16, and 0.16, respectively, and the p-values were less than 0.001, so H1-1, H1-2, and H1-3 were established; the regression coefficients between PEE as well as NEE and residents’ VCB were 0.25 and 0.22, respectively, and the p-values were less than 0.001, so H2-1 and H2-2 were valid.

#### 4.3.2. The Mediating Role of EE in the Relationship between EC and VCB

To more precisely examine the mediating role of PEE and NEE between residents’ EC and VCB, we used SPSS macro INDIRECT in combination with the Bootstrap methods to verify the significance of some of the mediating effects [45]. As required, EC was placed in the independent variable position, PEE and NEE were placed in the mediating variable position, VCB was placed in the dependent variable position, and the “Bootstrap Samples” value was set to 5000 with a confidence level of 95%. The results are shown in Table 8. The confidence intervals for both the mediating variables did not contain 0 (Table 8), so both PEE and NEE partially mediated the relationship between EC and VCB. In addition, the interval of the difference in the strength of the mediating effect between the two included 0, indicating that there was no significant difference. As a result, H3-1, H3-2, and H3 were verified.

## 5. Discussion

This study aimed to explore the influence of EC and EE on residents’ VCB and the mediating role of EE. The results showed that both EC and EE had a significant positive effect on residents’ VCB, suggesting that urban residents who had a certain level of awareness of environmental issues or who expressed a strong emotion about environmental issues were more likely to take the initiative to reduce carbon. In addition, we found that residents’ EC had a much higher impact on VCB than EE. However, most existing studies have concluded that EE significantly impacts pro-environmental behavior more than EC [11]. This paper contradicted the results of previous studies. On the one hand, it was because the definition of the concepts of cognition and emotion and the classification of their dimensions differed from previous studies. On the other hand, it may be because emotion is a continuous and stable state, which is the result of a continuous evolution of cognition, and some of its effects on residents’ voluntary carbon reduction may not be reflected immediately and are delayed.

### 5.1. Correlation of EC and VCB

The three dimensions of EC (CEI, CCK and CIR) significantly positively impacted residents’ VCB. It showed that urban residents’ awareness of environmental issues, knowledge of carbon reduction, and awareness of individual responsibility were enhanced, which would help to increase their enthusiasm for carbon reduction.

For CEI, the significant positive impact of CEI on VCB showed that when residents realized the problem of environmental degradation, they would take the initiative to avoid “high carbon” behaviors in their lives. Residents who were concerned and cared about environmental issues were more likely to participate in environmental protection activities. The more individuals are aware of the pollution in their current living environment, the more likely they are to voluntarily reduce their carbon footprint for reasons of health and profitability.

For CCK, the study results showed that CCK positively influenced VCB and that individuals’ knowledge of carbon reduction was an essential basis for engaging in VCB. However, it was noted that a wealth of knowledge did not necessarily promote low-carbon behavior in individuals and that increased environmental knowledge did not necessarily lead to non-environmental behavior change [46]. The inconsistency in subsequent environmental behavior caused by the perception of environmental knowledge is probably due to the fact that once individuals have some environmental knowledge, they will re-examine the current state of their environment and will be reluctant to engage in pro-environmental behavior when they find deficiencies.

For CIR, we found that it had a significant positive impact on residents’ VCB. However, earlier research showed that people usually attribute responsibility for protecting the environment and conserving resources to the government or related organizations and that the perception of green responsibility was low [47]. As society has evolved, scholars have found a correlation between an individual sense of responsibility and pro-environmental behavior [48]. Low-carbon responsibility perception influences people’s behavioral preferences, and when this awareness is derived from an individual’s own practice, individuals’ awareness and behavior will remain consistent. For urban residents, implementing carbon-reduction behaviors can gain a sense of individual participation and authenticity. Therefore, the awareness of individual responsibility can significantly affect the VCB of residents.

### 5.2. Correlation of EE and VCB

The result showed that both dimensions of EE (PEE and NEE) significantly positively affected VCB. It was similar to the findings recommended by previous studies [49,50]. EE is an individual’s attitudinal experience of VCB, and the stronger the emotional experience, the more inclined one is to engage in VCB. The positive psychological states of individual residents, such as love, praise, and pride for their own or others’ carbon-reduction behaviors, help to reproduce carbon-reduction behaviors. Residents’ guilt or disgust about their own or others’ high-carbon lifestyle behaviors may also contribute to their subsequent active choice to reduce carbon. The study of Bamberg and Moser [51] confirmed this point. In addition, we found that compared with PEE, NEE could affect residents’ VCB to a greater extent. It may be due to the fact that NEE can bring greater stimulation to individual residents and last longer, which is more helpful in stimulating residents’ VCB.

### 5.3. The Mediating Effect of EE between EC and VCB

This paper found that EE mediated the relationship between EC and VCB, which was similar to previous scholars’ findings [34,52]. Although EC has a direct impact on VCB, the emotional component is essential if individual residents are to sustain their VCB. In addition, we found that PEE and NEE played a mediating role in EC and VCB, respectively, and there was no significant difference in the strength of the mediating effect of PEE and NEE. It suggested that both PEE and NEE played an important role in transforming residents’ environmental perception into their voluntary implementation of carbon-reduction behaviors.

## 6. Conclusions and Policy Implications

This paper used the multiple linear regression and bootstrap analysis methods to explore the impact mechanism of urban residents’ individual EC and EE on their VCB. In general, residents’ EC and EE had a significant positive impact on residents’ VCB. The three dimensions of EC (CEI, CCK, and CIR) and the two dimensions of EE (PNN and NEE) all had a significant positive effect on VCB. In addition, this paper also verified the mediating effect of EE between EC and VCB, and there was no significant difference in the strength of the mediating effect between PEE and NEE.

This study has both theoretical and practical contributions. Firstly, most published studies have explored the relationship with pro-environmental behavior from an emotional or cognitive perspective, but there was a lack of research that considers the voluntary aspects of carbon reduction from an emotional and cognitive perspective, which this paper filled. This study confirmed the important positive role of EE and EC for VCB and the mediating role of EE between EC and VCB. Secondly, in terms of practical value, the results of this study have important guidance for society to promote the implementation of comprehensive residential voluntary carbon reduction.

The EC of urban residents can stimulate individuals’ EE, which in turn leads to their VCB. EC has a stronger positive impact on VCB than EE, so focusing on increasing urban residents’ knowledge of carbon reduction, awareness of environmental issues, and responsibility can help increase their motivation to reduce carbon. However, the EC and EE of residents cannot be formed by themselves but rely on the efforts of various stakeholders, such as the government and environmental protection organizations, to promote them through various efforts. Therefore, to avoid campaign-style “carbon reduction”, a long-term mechanism of carbon reduction must be established. We propose the following suggestions: (1) For social media, it is vital to strengthen publicity and education on the current state of environmental issues and carbon-reduction knowledge to raise residents’ CEI and CCK. At the same time, stories, music, and other forms should be used to stimulate residents’ feelings towards the environment. (2) For the government and environmental protection departments, it is necessary to establish a sound system of carbon-reduction-related institutions to stimulate and raise residents’ CIR and promote their active implementation of corresponding carbon-reduction responsibilities. (3) For enterprises and related technical departments, the information-sharing platform for carbon reduction can be built through organizational support and government subsidies, which will serve as an incentive and supervision for residents, mobilize residents’ emotions and awareness of carbon reduction, and enhance their motivation to reduce carbon.

## Figures and Tables

**Table 1:** Sample characteristics.

Measure		Sample Size (n)	Percentage (%)
Gender	Male	501	51.23
	Female	477	48.77
Age	18–25	159	16.26
	26–35	468	47.85
	36–45	267	27.30
	46–60	75	7.67
	>60	9	0.92
Education	High school and below	253	25.87
	College degree	298	30.47
	Bachelor’s degree	336	34.36
	Master’s degree and above	91	9.30
Income	≤Ұ2000	122	12.48
	Ұ2000–Ұ6000	352	35.99
	Ұ6000–Ұ10,000	405	41.41
	>Ұ10,000	99	10.12

**Table 2:** Reliability and convergent validity analysis.

Variable	Item	Estimate	AVE	CR
VCB	I don’t pay much attention to my environmental behavior	0.57	0.42	0.84
	I will advise people to implement carbon-reduction behavior	0.66		
	I think protecting the environment can improve my image	0.65		
	I think not actively reducing carbon will be condemned	0.64		
	I can actively participate in low-carbon welfare activities	0.68		
	I will initiate suggestions on environmental issues to the authorities	0.67		
	I actively participate in meetings related to a low-carbon life	0.68		
CEI	Fire power accounts for over 70% of China’s overall power output	0.71	0.47	0.78
	China’s energy of unit GDP is far higher than the global level	0.63		
	China’s CO_2_ emission of unit GDP is higher than developed nations	0.67		
	Environmental investment as a proportion of GNP is low	0.73		
CCK	10% of household energy consumption from appliance standby	0.73	0.46	0.77
	Refrigerators cool best at 80% capacity	0.72		
	Increasing the air conditioning temperature saves electricity	0.55		
	A washer with too few or too many clothes can increase power use	0.69		
CIR	I barely pay attention to climate change	0.71	0.51	0.76
	I think carbon reduction is the government’s responsibility	0.72		
	I don’t think carbon emission reduction has much to do with me	0.72		
PEE	I am delighted and proud to be involved in carbon reduction	0.65	0.44	0.7
	I respect and encourage others to reduce their carbon footprint	0.64		
	I enjoy being close to nature	0.69		
NEE	I would feel guilty and sad for acting environmentally friendly	0.68	0.43	0.69
	I am disgusted by the non-environmental behavior of others.	0.67		
	It pains me to see nature destroyed	0.62		

AVE: average variance extraction, CR: combined reliability.

**Table 3:** Reliability indices.

Variable	VCB	CEI	CCK	CIR	PEE	NEE
Cronbach’s α	0.84	0.78	0.77	0.76	0.70	0.69

**Table 4:** Confirmatory factor analysis of discriminant validity.

Model	χ^2^	df	χ^2^/df	RMSEA	GFI	CFI	Model Comparison	∆χ^2^	∆df
1. Original	593.456	237	2.504	0.039	0.950	0.962			
2. Five-factor	624.913	242	2.582	0.040	0.947	0.959	2 vs. 1	31.457 ***	5
3. Four-factor	636.718	246	2.588	0.040	0.946	0.958	3 vs. 1	43.262 ***	9
4. Three-factor	879.440	249	3.532	0.051	0.922	0.932	4 vs. 1	285.984 ***	12
5. Two-factor	1490.324	251	5.938	0.071	0.849	0.866	5 vs. 1	896.868 ***	14
6. One-factor	1559.496	252	6.188	0.073	0.844	0.859	6 vs. 1	966.040 ***	15

*** *p* < 0.001. Model 2: VCB, CEI + CCK, CIR, PEE, NEE. Model 3: VCB, CEI + CCK, CIR, PEE + NEE. Model 4: VCB, CEI + CCK, CIR + PEE + NEE. Model 5: VCB, CEI + CCK + CIR + PEE + NEE. Model 6: VCB + CEI + CCK + CIR + PEE + NEE.

**Table 5:** Descriptive statistics and correlation matrix for all variables.

	M	SD	1	2	3	4	5	6
1. VCB	3.683	0.849	1					
2. CEI	3.590	0.911	0.644 **	1				
3. CCK	3.569	0.911	0.668 **	0.709 **	1			
4. CIR	3.767	0.943	0.603 **	0.403 **	0.407 **	1		
5. PEE	3.836	0.878	0.649 **	0.515 **	0.496 **	0.482 **	1	
6. NEE	3.828	0.872	0.665 **	0.534 **	0.529 **	0.551 **	0.693 **	1

** *p* < 0.01, M: mean; SD: standard deviation.

**Table 6:** Difference analysis of the scale scores of the present study.

Group	Statistic	VCB	CEI	CCK	CIR	PEE	NEE
Gender	Male	3.648 ± 0.885	3.621 ± 0.899	3.566 ± 0.906	3.699 ± 0.975	3.806 ± 0.867	3.785 ± 0.881
	Female	3.720 ± 0.809	3.557 ± 0.923	3.571 ± 0.917	3.837 ± 0.905	3.868 ± 0.89	3.874 ± 0.861
	T	−1.315	1.101	−0.093	−2.29 *	−1.095	−1.587
Age	18–25	3.483 ± 0.761	3.45 ± 0.926	3.346 ± 0.948	3.786 ± 0.91	3.857 ± 0.773	3.883 ± 0.806
	26–35	3.703 ± 0.876	3.617 ± 0.916	3.553 ± 0.902	3.733 ± 0.952	3.855 ± 0.867	3.827 ± 0.868
	36–45	3.771 ± 0.843	3.659 ± 0.875	3.696 ± 0.904	3.795 ± 0.964	3.81 ± 0.936	3.823 ± 0.898
	46–60	3.709 ± 0.767	3.513 ± 0.926	3.72 ± 0.817	3.844 ± 0.896	3.827 ± 0.943	3.769 ± 0.91
	≥61	3.381 ± 1.237	3.25 ± 1.083	3.278 ± 0.988	3.667 ± 0.928	3.333 ± 0.928	3.593 ± 1.176
	F	3.324 *	1.885	4.519 **	0.380	0.877	0.408
Income	≤2000	3.46 ± 0.8	3.334 ± 0.993	3.262 ± 1.005	3.768 ± 0.954	3.836 ± 0.811	3.907 ± 0.841
	2000–6000	3.761 ± 0.832	3.634 ± 0.899	3.62 ± 0.897	3.806 ± 0.89	3.857 ± 0.885	3.89 ± 0.821
	6000–10,000	3.689 ± 0.862	3.612 ± 0.892	3.604 ± 0.895	3.727 ± 0.974	3.805 ± 0.902	3.765 ± 0.904
	≥10,000	3.661 ± 0.88	3.659 ± 0.881	3.619 ± 0.844	3.788 ± 0.996	3.892 ± 0.845	3.768 ± 0.937
	F	3.840 **	3.785 *	5.340 **	0.460	0.370	1.789

* *p* < 0.05, ** *p* < 0.01.

**Table 7:** Regression analysis.

Variable	Model 1			Model 2		
	β	t	Sig.	β	t	Sig.
(Constant)	0.073	0.873	0.383	0.067	0.798	0.425
EC	0.616	21.522	0.000			
EE	0.357	13.300	0.000			
CEI				0.197	7.641	0.000
CCK				0.158	5.741	0.000
CIR				0.156	6.117	0.000
PEE				0.246	9.725	0.000
NEE				0.217	10.636	0.000
R^2^	0.668			0.670		
Adjusted R^2^	0.668			0.668		
F	981.694			394.463		
Significance	0.000			0.000		

**Table 8:** Regressions testing EE as a mediator in the relationship between EC and VCB.

	Effect	SE	LLCI	ULCI	Effectiveness Ratio
Total effect	0.274	0.021	0.234	0.317	45.69%
PEE	0.127	0.018	0.093	0.163	21.21%
NEE	0.147	0.017	0.114	0.182	24.50%
PEE-NEE	0.020	0.028	−0.036	0.075	-

SE: standard error; LLCI: lower limit confidence interval; ULCI: upper limit confidence interval.

## Data Availability

Some or all data and models that support the findings of this study are available from the corresponding author upon reasonable request.
